# LipL41 and LigA/LigB Gene Silencing on a LipL32 Knockout *Leptospira interrogans* Reveals the Impact of Multiple Mutations on Virulence

**DOI:** 10.3390/pathogens12101191

**Published:** 2023-09-24

**Authors:** Luis Guilherme V. Fernandes, Bruno B. Foltran, Aline F. Teixeira, Ana Lucia Tabet Oller Nascimento

**Affiliations:** 1 Laboratório de Desenvolvimento de Vacinas, Instituto Butantan, Avenida Vital Brazil, 1500, São Paulo 05503-900, SP, Brazil; bruno.foltran.esib@esib.butantan.gov.br (B.B.F.); aline.rteixeira@butantan.gov.br (A.F.T.); 2 Programa de Pos-Graduacao Interunidades em Biotecnologia, Instituto de Ciencias Biomedicas, São Paulo 05508-900, SP, Brazil

**Keywords:** *Leptospira*, knockout, knockdown, CRISPR-interference, LipL32, LipL41, Lig proteins, multiple mutations

## Abstract

Leptospirosis is a global zoonosis caused by pathogenic bacteria of the genus *Leptospira*. The application of the CRISPR/Cas9 system has facilitated the generation of mutants and subsequent evaluation of phenotypes. Since DNA breaks induced by RNA-guided Cas9 nuclease are lethal to *Leptospira*, different methodologies were implemented to overcome this limitation. Initially, CRISPR interference (CRISPRi) was employed to create knockdown mutants, utilizing a catalytically inactive Cas9 (dCas9). Subsequently, the co-expression of CRISPR/Cas9 and a DNA repair system from *Mycobacterium smegmatis* enabled the generation of scarless knockout mutants. We eliminated plasmids from the *lipL32* knockout *L. interrogans* strain and further achieved multiple gene mutations via gene silencing in this knockout background. Strains lacking both LipL41 and LipL32 and LigA, LigB, and LipL32, were evaluated. The absence of proteins LipL32 and LipL41 had no effect on leptospiral virulence. On the other hand, mutants lacking LigA, LigB, and LipL32 were unable to cause acute disease. The expanded apparatus for genetic manipulation of pathogenic leptospires via the CRISPR/Cas9 system has allowed the evaluation of multiple mutations upon leptospiral virulence. This work shows that LipL32 and LipL41 are not required for acute disease and consolidates LigA and LigB proteins as virulence factors.

## 1. Introduction

Leptospirosis is a global zoonotic disease caused by pathogenic species of the genus *Leptospira*. This neglected disease is widespread and affects both reservoir and incidental hosts via contact with urine and other biological fluids of infected animals, or indirectly via exposure to contaminated water or soil [1,2]. With more than one million cases and almost 60,000 human deaths per year worldwide, leptospirosis is a serious public health concern [3,4]. However, due to a lack of surveillance, rapid diagnostic tests, and notification in high-risk countries, mortality and incidence statistics are likely underestimated [5,6].

The symptoms of human leptospirosis can range from flu-like symptoms such as fever, myalgia, and headache to more severe forms such as Weil’s disease, which represents 5–15% of reported cases and can be potentially fatal [7]. Leptospirosis-associated pulmonary hemorrhage syndrome (LPHS) is an even more serious form of the disease, with a fatality rate of over 50% [8,9].

Leptospirosis is well documented in various animal species, including cattle, swine, sheep, goats, horses, and dogs [10,11,12]. Bovine leptospirosis poses a significant economic loss due to abortions, stillbirths, poor reproductive performance, and loss of milk production, leading to failure to thrive and stillbirth phenotypes in calves [10,13,14].

The recent application of the CRISPR/Cas9 system to *Leptospira* spp. has expanded our knowledge of leptospiral biology, host–pathogen interaction, and above all, virulence factors upon observation of avirulent mutants in animal models [15,16,17,18]. The search for virulence factor has been sped up by CRISPR interference-mediated gene silencing. This innovative genetic tool relies on the expression of a programmable DNA-binding protein, which is catalytically inactive Cas9 (dCas9) and a single-guide RNA [15,19].

Thus far, leptospires unable to cause lethal disease in hamsters include knockdown strains for LipL21 [20] and both LigA and LigB [17]; this latter phenotype is doable by only one sgRNA capable of pairing to the conserved nucleotide region of both *ligA* and *ligB* genes. Multiple silencing by tailoring plasmids with in-tandem sgRNA cassettes is feasible, but plasmid instability has been reported [20]. In addition, the silencing of LipL32, the major outer membrane protein of pathogenic *Leptospira* spp., resulted in more virulent mutants [17,20], with substantial upregulation of other proteins that are potential virulence factors, including LigA and LigB.

More recently, a knockout mutant for LipL32 was obtained via the concurrent expression of the CRISPR/Cas9 system and non-homologous end-joining DNA repair machinery from *Mycobacterium smegmatis*, since double-strand breaks induced by Cas9 are lethal to *Leptospira* spp. A plasmid coding for Cas9, sgRNA, and mycobacterial LigD and Ku proteins allowed the recovery of LipL32 scarless knockout mutants, with deletions in the junctional site. Due to the permanent status of these mutations, the plasmid can be eliminated, resulting in markerless knockout strains [18]. Plasmid elimination offers a foundation for including additional mutations and/or gene silencing, enabling the investigation of the concurrent impact of leptospiral proteins upon virulence and other biological processes.

Since functional redundancy is commonly observed in *Leptospira* pathogenesis, we aimed to investigate the impact of multiple mutations on *L. interrogans* virulence. To achieve this, we cured the plasmid in the *lipL32* knockout leptospires, resulting in a markerless mutant strain. Then, we used CRISPRi-mediated gene silencing on the LipL32 knockout mutant genetic background and successfully generated mutants with abolished expression of LipL41 and LipL32, as well as mutants with abolished expression of LipL32, LigA, and LigB. Animal infection experiments were conducted, revealing for the first time that leptospires lacking both major outer membrane proteins LipL32 and LipL41 retained the ability to cause lethal disease in hamsters. Notably, despite the anticipated increase in virulence due to the absence of LipL32, the introduction of further silencing targeting LigA and LigB led to leptospires that lost their capacity to cause lethal disease. This finding strengthens the major role of Lig proteins in virulence.

## 2. Materials and Methods

### 2.1. Bacterial Strains and Plasmids

Virulent low-passage clones of a LipL32 mutant of *L. interrogans* serovar Copenhageni strain Fiocruz L1-130 obtained by the CRISPR/Cas9-NHEJ strategy [18] were cultured in EMJH medium [21] and 1% rabbit serum. Media plates were prepared with 1.2% Noble agar (Difco). For colony recovery, spectinomycin (40 μg/mL) was added to the plates. *E. coli* strain β2163 auxotrophic for diaminopimelic acid (DAP) [22,23] was used as conjugation donor cells and was grown in Lysogeny Broth (LB, Difco, Franklin Lakes, NJ) medium supplemented with DAP (0.3 mM, Sigma, St. Louis, MO). Plasmids pMaOri.dCas9 containing sgRNA cassettes targeting *lipL41* [20] and both *ligA* and *ligB* [16] were used for silencing.

### 2.2. Plasmid Curing on lipL32 Knockout L. interrogans

Cells of *L. interrogans* serovar Copenhageni LipL32 knockout for LipL32 designated as clones “a” and “b” were previously obtained by Fernandes et al. [18] and grown in liquid EMJH medium without antibiotic selection for plasmid elimination for 5 passages. Then, to avoid virulence loss, both clones were intraperitoneally inoculated in hamsters (n = 2) and animals were monitored daily for clinical signs of acute leptospirosis, including weight loss (>10%), blood on paws/nose/urogenital tract, lethargy, microphthalmia, hunched posture, dehydration, and prostration. Upon reaching endpoints, animals were humanely euthanized, and their kidney and liver were aseptically extracted, macerated, and inoculated in EMJH medium plus 5-fluorouracil (5-FU). Cultures were monitored daily and when cell densities reached around 10^8^ leptospires/mL; they were harvested via centrifugation (10,000× *g*, 15 min), washed twice with PBS, and cell lysates were prepared for protein separation via SDS-PAGE. Proteins were electrotransferred in semi-dry equipment and recovered mutants were re-validated by immunoblotting with anti-LipL32 and anti-LipL41 polyclonal antibodies (1:40,000).

Leptospires were brought to 10^3^ cell/mL and 100 µL of suspensions were used to inoculate EMJH plates for individual colony isolation. After 3 weeks, 10 colonies from both clones “a” and “b” were selected, and leptospires were released from the agar by extensive pipetting and grown in liquid EMJH with or without spectinomycin. Colonies that grew exclusively in media without antibiotic selection were inferred as plasmid-free and out of those, 5 cultures were selected for DNA extraction and validation via PCR with primers to the *lipL32* CDS and pMaOri2 [18] to the plasmid sequence. Cultures that were PCR-negative for the plasmid were further propagated and used for gene silencing via CRISPRi.

### 2.3. Bacterial Conjugation

Recombinant conjugative *E. coli* strain β2163 was used to deliver the plasmids to the *lipL32* null mutant of *L. interrogans* by conjugation, as previously described [16,23]. *E. coli* cells containing each of the desired plasmids were diluted 1:100 in fresh LB medium containing DAP until an optical density of 0.2–0.4 at 420 nm was reached. Then, they were mixed at a 1:1 cell proportion with *L. interrogans* cells. Leptospires were grown in EMJH medium until they reached an OD420 nm of 0.2–0.4. Cells were concentrated on a 25 mm diameter, 0.1 µm pore size, mixed cellulose esters membrane (Millipore, VCWP02500, Burlington, MA) using a filtration apparatus connected to a vacuum pump. The membranes were placed on EMJH plus DAP plates and incubated at 30 °C for 24 h. The cells were recovered from the filters and seeded on EMJH plates containing spectinomycin for the selection of recombinant leptospires. After approximately three weeks of incubation at 30 °C, colonies were retrieved from plates and examined under a microscope to confirm live and motile cells. The cells were then transferred into liquid EMJH plus spectinomycin for further experiments.

### 2.4. Electrophoresis and Immunoblotting

*L. interrogans* cultures were grown until the mid to late-log phase, recovered via centrifugation (10,000× *g*, 15 min), and the resulting pellet was washed twice with PBS. Whole-cell lysates were processed for SDS-PAGE on 12% acrylamide gels (BioRad, Hercules, CA, USA). For immunoblotting, proteins were electrotransferred onto nitrocellulose membranes (Hybond ECL; GE Healthcare, Chicago, IL, USA) by semidry transfer. Membranes were blocked in PBS containing 10% non-fat dried milk for 1 h and then incubated with the indicated primary antibody diluted in the blocking buffer for 1 h at room temperature. Membranes were washed 3 times with PBS 0.05% Tween 20 (PBS-T) and incubated with horseradish peroxidase-conjugated secondary anti-mouse IgG (1:5000) antibody in blocking buffer for 1 h at room temperature. After six washing steps, the detection of antigen reactivity was revealed using SuperSignalTM West Dura Extended Duration Substrate (Thermo Scientific, Waltham, MA, USA), and the luminescence generated was detected with the aid of an Amersham Imager 600 (GE).

### 2.5. Animal Ethics Statement

The Ethics Committee for Animal Research of Instituto Butantan approved the use of the animals involved in these studies under protocol number 8790290422. The Committee on Animal Research adopts the guidelines of the Brazilian College of Animal Experimentation.

### 2.6. Animal Infection

Knockdown mutants for LipL41 (pMaOri.dCas9sgRNAlipL41) or both LigA and LigB (pMaOri.dCas9sgRNAligAB) in the LipL32 knockout background (KO32) were cultured to mid–late log phase in EMJH medium at 29 °C. Four- to six-week-old male Syrian gold hamsters (*Mesocricetus auratus*) were acclimated to the facility a week prior to the challenge and segregated into boxes of 4 or 5 animals with similar general weight averages. The weight of each animal was considered 100% on the day of infection. Animals were monitored daily and always had ad libitum access to food and water. Groups were inoculated intraperitoneally with 5 × 10^7^ distinct leptospiral mutants at the same in vitro passage. Each animal was monitored daily and weighed for clinical signs of acute leptospirosis. Animals were humanely euthanized when weight loss (>10%) and/or additional clinical signs were observed, including blood on paws/nose/urogenital tract, lethargy, microphthalmia, hunched posture, dehydration, and prostration. One kidney and a liver section were harvested and immediately macerated in 5 mL of EMJH medium containing 5-FU. Fifty µL of macerates were used to inoculate 5 mL of EMJH medium plus 5-FU with spectinomycin (40 µg/mL), to isolate mutant bacteria. High pMaOri.dCas9 backbone stability was demonstrated in vivo and in vitro, even in the absence of antibiotic selection [20]. Cultures were monitored daily via dark-field microscopy. An additional section of the kidney and liver were harvested for DNA extraction and bacterial burden quantification.

Endpoints of animals inoculated with KO32 knockdown strains were compared to those displayed by animals infected with KO32 *L. interrogans* containing empty pMaOri.dCas9 plasmids by the log-rank test; and *p* < 0.05 was considered statistically significant.

### 2.7. Quantification of Leptospires in Target Organs

Kidney and liver tissue (around 15 mg) were used for DNA isolation using the DNeasy DNA Blood and Tissue isolation kit (Qiagen, Germantown, MD). The concentration curve of leptospiral genomic equivalent (GEq) was prepared with genomic DNA from wild-type *L. interrogans*. The bacterial load was quantified via quantitative PCR assay using a CFX96 Real-Time System (Bio-Rad, Hercules, CA, USA). The lipL32 gene was amplified in the samples (5 µL template/well) using primers qLipL32F (5′AAGGATCTTTCGTTGCATCT3′, pairing to nt 668–687) and qLipL32R (5′TTACTTAGTCGCGTCAGAAG3′, pairing to nt 800–819), which are not affected by the *lipL32* mutation in the clone used for silencing (deletion from nt 59 to 68 in *lipL32* CDS); the 152-bp amplicon was detected using SYBR Green PCR Master Mix (Applied Biosystems, Foster City, CA, USA). The total volume of PCR reactions was 20 µL containing 400 nM of each primer. The amplification protocol consisted of 10 min at 95 °C, followed by 40 cycles of amplification (95 °C for 15 s and 60 °C for 1 min). A result was considered negative if the Ct was greater than 40. The concentration of leptospires (GEq per gram of tissue) was calculated based on the standard curve equation. Statistical differences between groups of interest were determined using a one-tailed Mann–Whitney non-parametric test, and a *p* < 0.05 was considered statistically significant.

## 3. Results

### 3.1. Plasmid Curing in the LipL32 Mutant L. interrogans

LipL32 knockout mutants (KO32) of *L. interrogans* serovar Copenhageni strain Fiocruz L1-130 were obtained previously via the concurrent expression of the CRISPR/Cas9 system and the M. smegmatis NHEJ machinery protein LigD and Ku. Two clones were obtained, named clones “a” and “b”, in which abolished LipL32 expressions were due to a 10 and 345 bp deletion in the junctional site, respectively [18]. However, the presence of the pMaOriCas9NHEJ plasmid was still detectable in cultures, exemplified by the predominant presence of spectinomycin-resistant cells. The pMaOri backbone is relatively stable when bacterial growth occurs in the absence of antibiotics, but a gradual loss in cell population can be observed after several in vitro passages [15,24]. In this regard, cultures from clones “a” and “b” were propagated in EMJH media without spectinomycin for five passages, and then, to avoid virulence attenuation, cells were used to infect hamsters for leptospiral isolation in target organs.

**Figure 1:**
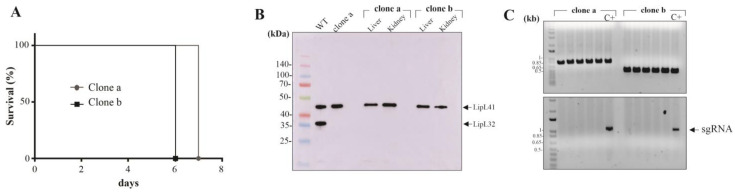
Plasmid curing in virulent lipL32 knockout strains. (**A**) Hamsters were infected with 10^8^ cells of L. interrogans strain Fiocruz L1-130 knockout for LipL32 protein (KO32) and animals were monitored daily for clinical signs of acute leptospirosis. (**B**) Upon reaching the endpoint, animals were humanely euthanized, and their kidney and liver were aseptically extracted, macerated, and inoculated in EMJH medium plus 5-FU. After growth, leptospires were harvested from the media and assessed by immunoblotting with anti-LipL32 and anti-LipL41 polyclonal antibodies. (**C**) For selecting plasmid-free isolates, leptospires were brought to 10^3^ cell/mL, and 100 µL of suspensions were used to inoculated EMJH plates for individual colony isolation. Ten colonies from both clones “a” and “b” were selected and grown in liquid EMJH with or without spectinomycin. Colonies that grew exclusively in media without antibiotic selection were inferred as plasmid-free, their DNA extracted and further validated by PCR with primers to the lipL32 CDS (upper panel) and the plasmid sequence (lower panel). As a positive control, DNA from cultures grown in the presence of spectinomycin was used.

Animals who displayed signals of severe leptospirosis, including weight loss (>10%), blood in the urogenital tract and prostration, and were humanely euthanized (Figure 1A), and their liver and kidneys were extracted for bacterial isolation in EMJH media. After growth, mid-log phase cells were recovered and processed for immunoblotting analysis and demonstration of LipL32 abolished expression (Figure 1B). Cultures were diluted and seeded in EMJH plates without spectinomycin, and ten colonies from each clone were randomly picked and inoculated in EMJH liquid media with or without spectinomycin. Those clones that displayed exclusive growth in media without antibiotics were considered plasmid-free, a feature further supported by PCR with primers targeting the lipL32 gene and sgRNA cassette in the plasmids. As seen in Figure 1C, no amplification of the sgRNA cassette was observed in the cultures selected from clones “a” and “b” (lower panel), in contrast to the positive control comprised of DNA extracted from cell growth in the presence of spectinomycin. As expected, the *lipL32* amplification was observed as smaller amplicons in clone “b”, (upper panel) due to its larger deleted fragment.

### 3.2. Gene Silencing by CRISPRi in KO32 L. interrogans

**Figure 2:**
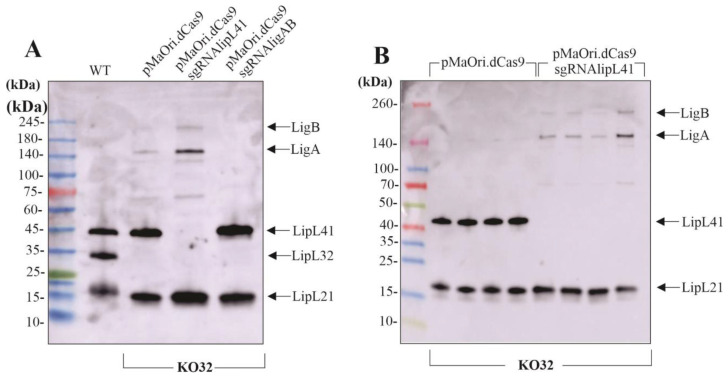
Validation of mutant leptospires. (**A**) Cultures of mutant L. interrogans were centrifuged and protein samples were processed for SDS-PAGE. Proteins were electrotransferred onto nitrocellulose membranes, which were blocked PBS containing 10% non-fat dried milk for 1 h and then incubated with anti-LipL32 (1:40,000), LipL41 (1:40,000), anti-LipL21 (1:20,000) and anti-Lig (1:1000) for 1 h at room temperature. Membranes were then washed and incubated with horseradish peroxidase-conjugated secondary anti-mouse IgG (1:5000) antibody in a blocking buffer for 1 h at room temperature. (**B**) Distinct clones of KO32 LipL41 knockdown mutant were evaluated regarding LigA and LigB upregulation.

Virulent, plasmid-free cells from KO32 clone “a” were selected for bacterial conjugation with *E. coli* harboring the CRISPRi plasmids pMaOri.dCas9sgRNAlipL41 and sgRNAligAB. A plasmid without a sgRNA cassette was also used for control in the downstream infection experiments. Colonies grew in approximately 3 weeks and were recovered from plates for growth in media with spectinomycin. Mid-log phase cells were harvested and whole-cell extracts were evaluated by immunoblotting with polyclonal serum anti-LipL21, anti-LipL32, anti-LipL41, and anti-LigAB raised against the conserved region of Lig proteins (Figure 2A). It is worth noticing that LigA and LigB expression is upregulated when LipL32 is absent in comparison to the wild-type control, a behavior that was previously demonstrated [17]. When KO32 cells contained pMaOridCas9 plasmid alone, only the LipL32 abolished expression was observed; LipL41 protein was silenced when plasmid included the sgRNA targeting this specific gene, and interestingly, LigA and LigB proteins expression appears to be upregulated in this mutant to compensate the LipL41 loss. This phenomenon was observed throughout all immunoblots and clones tested (Figure 2B). As expected, LigA and LigB were silenced when leptospires contained the sgRNAligAB cassette. All these sgRNA constructions were previously utilized to silence the respective proteins in the wild-type strain [17,20]. It is worth mentioning that no difference in growth curves was observed among mutant strains.

### 3.3. Effect of Multiple Protein Mutation upon Leptospira Virulence

**Figure 3:**
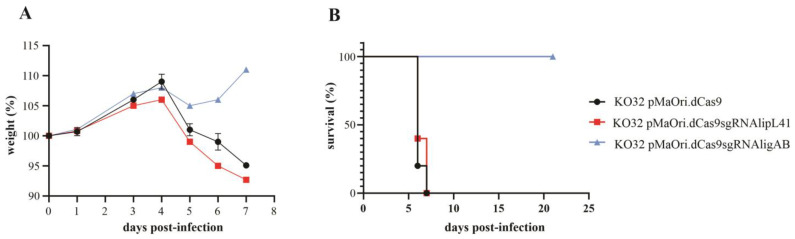
Virulence evaluation of mutant leptospires in animal model. Knockdown mutants for LipL41 (KO32 pMaOri.dCas9sgRNAlipL41) or both LigA and LigB (KO32 pMaOri.dCas9sgRNAligAB) in the LipL32 knockout background (KO32) at the same passage were used to intraperitoneally infect groups of male Syrian gold hamsters (group n = 4 or 5). Each animal was monitored daily and weighed for clinical signs of acute leptospirosis (**A**). Animals were humanely euthanized when weight loss (>10%) and/or additional clinical signs were observed (**B**). Endpoints of animals inoculated with KO32 knockdown strains were compared to those displayed by animals infected with KO32 *L. interrogans* containing empty pMaOri.dCas9 plasmids by the log-rank test; and *p* < 0.05 was considered statistically significant.

LipL32 knocked out *L. interrogans* containing the pMaOri.dCas9 plasmid alone or with the sgRNA cassettes for silencing LipL41 or both LigA and LigB were used to intraperitoneally infect male hamsters (n = 5 for dCas9 and LipL41 mutant groups, and n = 4 for LigAB mutant group). Animals were monitored daily for weight loss and additional clinical signs. Animals infected with KO32 cells with no additional mutations (pMaOri.dCas9 containing cells) displayed prominent weight loss on day 5 (relative drop from 108.7 to 101.2%), with four animals reaching endpoint criteria on day 6 and the remaining one on day 7 (Figure 3A and Table 1). When both LipL32 and LipL41 were absent, leptospirals were still virulent, inducing lethal leptospirosis in all animals, whereas three animals were euthanized on day 6 and the remaining two on day 7 (Table 1). No difference in severity was observed between these two groups. The silencing of LigA and LigB resulted in avirulent mutants (*p* = 0.0035) since no animals reached endpoint criteria or displayed acute signs of leptospirosis (Figure 3A,B), with only a minor drop of average relative weight on day 5, followed by a recovery starting on day 6.

### 3.4. Bacterial Burden in Target Organs and Mutant Recovery

**Figure 4:**
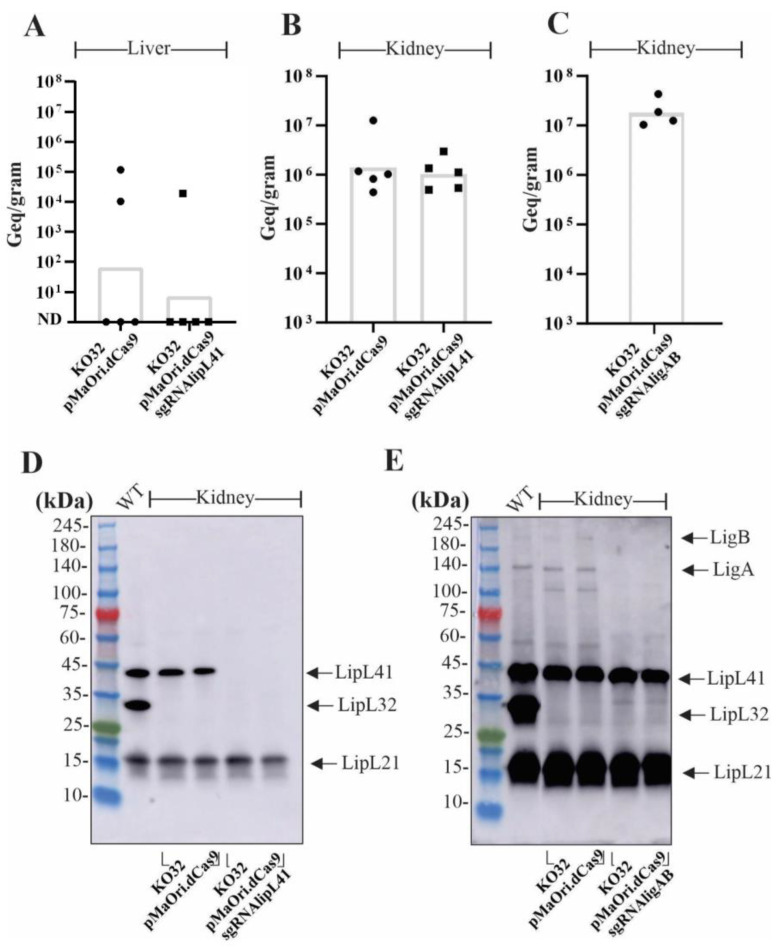
Quantification of bacterial load in target organs and mutant recovery. Liver (**A**) and kidney (**B**,**C**) tissue were used for DNA and bacterial load was quantified by a quantitative PCR assay with lipL32 primers. The concentration curve of leptospiral genomic equivalent (GEq) was prepared with genomic DNA from wild-type *L. interrogans*. The concentration of leptospires (GEq per gram of tissue) was calculated based on the standard curve equation. Statistical differences between groups of interest were determined by one-tailed Mann–Whitney non-parametric test and a *p* < 0.05 was considered statistically significant. Mutants recovered from the kidney homogenates in EMJH medium plus spectinomycin were evaluated by immunoblotting. In (**D**) mutants for LipL41 (KO32pMaOri.dCas9sgRNAlipL41) and (**E**) mutants for LigA and LigB (KO32 pMaOri.dCas9sgRNAligAB).

Upon reaching endpoints or by the end of the experiment, the kidneys and livers from infected animals were extracted for bacterial quantification via quantitative PCR and isolation of mutants via tissue homogenates’ inoculation in liquid media containing spectinomycin. Positive cultures from liver macerates were observed in only two animals from KO32 dCas9 control animals and only one from the KO32 LipL41 mutant-infected group. In these groups, the geometric mean of the bacterial load was 65 and 7 GEq per gram of liver, respectively (Figure 4A). Based on the established cutoff, three liver samples from KO32 dCas9 and four samples from the KO32 LipL41 mutant-infected group were negative for the presence of leptospiral DNA, which agrees with the negative culture results obtained with these same samples. All kidney cultures from KO32 dCas9 control and KO32 LipL41 mutant-infect animals were positive, and quantification of bacterial load showed a geometric mean of 1.4 × 10^6^ and 1.04 × 10^6^ GEq/g, respectively (Figure 4B).

Since the KO32 LigAB mutant leptospires were avirulent, animals infected with this strain survived until the experiment and therefore were euthanized on day 21, and their organs were aseptically removed; all kidney cultures were positive and displayed an average of 1.8 × 10^7^ GEq per gram of tissue (Figure 4C). No growth was observed in cultures inoculated with liver macerates from these animals.

Leptospires recovered from kidney cultures were evaluated regarding the maintenance of mutations by immunoblotting. As seen in Figure 4D,E, the assessment of two representative cultures from each group corroborates the isolation of the respective mutants.

## 4. Discussion

*L. interrogans*, the etiological agent of human leptospirosis, is well known for its functional plasticity and redundancy. Since whole genome sequences became available [25,26,27], researchers started a herculean effort to elucidate the role of several potential virulence factors and hypothetical proteins. With a genome almost four times larger than the ones from other spirochetes [28], it was no surprise when functional genomics studies revealed overlapping roles to distinct leptospiral recombinant proteins [29,30]. This scenario anticipated that the development of an effective subunit vaccine would not be an easy task.

Haake and colleagues [31] first characterized the LipL32 protein, which was expected to be relevant to *Leptospira* pathogenesis, reinforced by its high conservation, copy number, and exclusive presence in pathogenic strains. Further characterization of the recombinant LipL32 revealed its broad spectrum of host ligands [32,33]. This protein is found at 38,000 copies/cell in *L. interrogans* [34] and is estimated to account for almost 75% of the OM proteome [35].

Ever since genetic manipulation of pathogenic leptospires became available, LipL32 became an obvious target for mutagenesis; nonetheless, early studies with random transposon mutagenesis frustrated most expectations and showed that the LipL32 knockout mutant could still cause acute and choric disease [36]. As the genetic toolbox evolved, it was confirmed that a knockdown mutant for LipL32 not only preserved its virulence in the hamster model but was augmented [17].

The recent application of the CRISPR/Cas9 system in confluence to the NHEJ machinery from *M. smegmatis* allowed the recovery of distinct knockout scarless clones of LipL32 [18]. Here, both clones were confirmed to still be virulent in the hamster model, and subsequently, to mutant isolation from kidneys and livers, plasmid-free strains could be obtained, permitting the delivery of CRISPRi plasmids for gene silencing in this LipL32 knockout genetic background. It is noteworthy to acknowledge that prior genetic manipulation methods used to generate knockout strains in *Leptospira* spp. involved the integration of an antibiotic resistance cassette into the genome, which hindered its subsequent removal. Our achievement of plasmid elimination and the subsequent creation of a markerless knockout mutant represents a highly promising advancement. This breakthrough could potentially pave the path towards the development of live attenuated vaccines or enhanced bacterins.

LipL41 is an abundant lipoprotein of pathogenic leptospires and requires the expression of a partner protein called Lep for its efficient and stable expression. A transposon mutant for LipL41 was still virulent in an animal model [37] and more recently, a CRISPRi-generated knockdown mutant was shown to be still able to cause acute disease in hamsters [20], although a slight attenuation in the symptoms was observed by the authors. LipL41 was found to be the major OM protein in the LipL32 transposon mutant [36], making it the second most abundant OM protein in the wild-type strain.

Here, we demonstrate for the first time that the complete absence of both LipL32 and LipL41 still results in virulent leptospires, with equivalent infection and endpoint kinetics and target organs loads. Based on previous results, it is expected that leptospires undergo a substantial proteomic chance to surpass the lack of its two most abundant proteins. In this sense, we could observe an apparent upregulation of both LigA and LigB in this mutant, in comparison to the knockout strain containing only the pMaOri.dCas9 plasmid, suggesting that these proteins are rapid responders to “fill the niche” left after protein mutation. It was previously demonstrated that LipL32 silencing in the wild-type L1-130 strain results in LigA and B protein upregulation, as per immunoblots observation and proteomic analysis [17].

Interestingly, both LigA and LigB are concomitantly required for acute leptospirosis, as knockdown leptospires in the wild-type background were unable to induce lethal infection in a hamster model. Knockdown mutants were obtained by distinct groups and methodology: Pappas and Picardeau [38] used Transcription Activator-like Effectors (TALE) targeting the promoter region of both genes in *L. interrogans* serovar Manilae, occasioning incomplete silencing. Then, Fernandes et al. [17] used CRISPRi to completely silence both genes in *L. interrogans* serovar Copenhageni, and although both works agree in the virulence attenuation phenotype in an animal model, only the CRISPRi mutants could still be recovered from target organs, most probably due to inoculum size and strain/serovar variability.

Since opposite phenotypes are observed by silencing of LipL32 or both LigA and LigB in the wild-type *L. interrogans* [17], we were interested in checking which phenotype would be outweighed in the absence of the three proteins. Remarkably, despite the absence of major LipL32, the silencing of both LigA and LigB resulted in the attenuation of virulence, with mutant bacteria unable to cause acute disease despite kidney colonization. These results reinforce the role of LigA and LigB as well-established virulence factors, and one of the probable mechanisms is the serum resistance conferred by these complement system-interacting proteins [16,39].

## 5. Conclusions

In conclusion, the expanded tools for genetic manipulation of pathogenic leptospires by the CRISPR/Cas9 system and its variants, used in conjunction, have allowed the evaluation of multiple mutations upon leptospiral virulence, showing that the two most abundant lipoproteins, LipL32 and LipL41, are not required for virulence. However, even with the expected increased virulence resulting from the lack of LipL32, additional silencing of LigA and LigB still results in avirulent bacteria that are unable to cause acute disease. This highlights the complexity of leptospiral virulence and emphasizes the need for continued research to fully understand the pathogenic mechanisms of this bacterium.

## Figures and Tables

**Table 1:** Animal experiment results.

LipL32 Knockout Strain Containing CRISPRi Plasmids					
	Endpoints/Total	Endpoint Day	Average Weight (%)		
			4	5	6
pMaOri.dCas9	5/5	6, 6, 6, 6, 7	108.7	101.2	98.8
pMaOri.dCas9sgRNAlipL41	5/5	6, 6, 6, 7, 7	105.6	98.99	95.2
pMaOri.dCas9sgRNAligAB	0/4	-	107.8	104.8	105.9

## Data Availability

The original contributions presented in the study are included in the article, further inquiries can be directed to the corresponding authors.
